# Bacterial meningitis in Africa

**DOI:** 10.3389/fneur.2023.822575

**Published:** 2023-02-14

**Authors:** Tatiana Barichello, Carlos Henrique Rocha Catalão, Ursula K. Rohlwink, Martijn van der Kuip, Dan Zaharie, Regan S. Solomons, Ronald van Toorn, Marceline Tutu van Furth, Rodrigo Hasbun, Federico Iovino, Vivian Ssonko Namale

**Affiliations:** ^1^Graduate Program in Health Sciences, University of Southern Santa Catarina (UNESC), Criciúma, SC, Brazil; ^2^Faillace Department of Psychiatry and Behavioral Sciences, McGovern Medical School, The University of Texas Health Science Center at Houston, Houston, TX, United States; ^3^Department of Neuroscience and Behavioral Science, Ribeirao Preto Medical School, University of São Paulo (USP), Ribeirao Preto, SP, Brazil; ^4^Pediatric Neurosurgery Unit, Red Cross War Memorial Children's Hospital, Cape Town, South Africa; ^5^Division of Neurosurgery, University of Cape Town, Cape Town, South Africa; ^6^Neuroscience Institute, University of Cape Town, Cape Town, South Africa; ^7^Department of Pediatric Infectious Diseases and Immunology, Amsterdam Infection and Immunity Institute, Amsterdam University Medical Centers, Vrije Universiteit, Amsterdam, Netherlands; ^8^Department of Anatomical Pathology, Faculty of Medicine and Health Sciences, Stellenbosch University, Cape Town, South Africa; ^9^National Health Laboratory Services, Tygerberg Hospital, Cape Town, South Africa; ^10^Department of Pediatric and Child Health, Faculty of Medicine and Health Sciences, Stellenbosch University, Cape Town, South Africa; ^11^Division of Infectious Diseases, Department of Internal Medicine, UT Health, McGovern Medical School, Houston, TX, United States; ^12^Department of Neuroscience, Karolinska Institutet, Stockholm, Sweden; ^13^Columbia University Irving Medical Center and New York Presbyterian Hospital, New York, NY, United States; ^14^Department of Paediatrics and Child Health, Makerere University College of Health Sciences, Kampala, Uganda

**Keywords:** bacterial, meningitis, Africa, pathophysiology, diagnosis, management

## Abstract

Bacterial meningitis differs globally, and the incidence and case fatality rates vary by region, country, pathogen, and age group; being a life-threatening disease with a high case fatality rate and long-term complications in low-income countries. Africa has the most significant prevalence of bacterial meningitis illness, and the outbreaks typically vary with the season and the geographic location, with a high incidence in the meningitis belt of the sub-Saharan area from Senegal to Ethiopia. *Streptococcus pneumoniae* (pneumococcus) and *Neisseria meningitidis* (meningococcus) are the main etiological agents of bacterial meningitis in adults and children above the age of one. *Streptococcus agalactiae* (group B Streptococcus)*, Escherichia coli*, and *Staphylococcus aureus* are neonatal meningitis's most common causal agents. Despite efforts to vaccinate against the most common causes of bacterial neuro-infections, bacterial meningitis remains a significant cause of mortality and morbidity in Africa, with children below 5 years bearing the heaviest disease burden. The factors attributed to this continued high disease burden include poor infrastructure, continued war, instability, and difficulty in diagnosis of bacterial neuro-infections leading to delay in treatment and hence high morbidity. Despite having the highest disease burden, there is a paucity of African data on bacterial meningitis. In this article, we discuss the common etiologies of bacterial neuroinfectious diseases, diagnosis and the interplay between microorganisms and the immune system, and the value of neuroimmune changes in diagnostics and therapeutics.

## Introduction

Bacterial meningitis is characterized by an inflammatory process in the meninges of the brain and spinal cord due to a bacterial infection. It causes significant mortality and morbidity worldwide, with the major burden of disease in Sub-Saharan Africa (1). A global burden of disease study showed that meningitis caused 318,000 deaths worldwide (4.5 per 100.000), resulting in 20,383 thousand years of life lost in 2016 (1). The incidence rates vary between 0.7–0.9 per 100.00 per year in the United States (US) and European countries, while in Africa, studies describe incidence rates between 0–40 per 100,000 per year (1, 2).

The epidemiology of bacterial meningitis varies widely by age (e.g., the higher incidence in neonates and elderly patients) (2). *Streptococcus agalactiae* (or Group B Streptococci) and *Escherichia coli* are the principal etiologies of neonatal meningitis (2). Recent epidemiological studies from Africa and the Netherlands show that between 2006 and 2014 of 1,412 episodes of community-acquired bacterial meningitis demonstrated that *Streptococcus pneumoniae, Neisseria meningitidis*, and *Listeria monocytogenes* accounted for 51, 37, and 4% of cases, respectively (3). *S. pneumoniae* and *N. meningitidis* cause up to 90% of cases in infants and children.

There are significant geographical differences in the epidemiology of bacterial meningitis worldwide. Sub-Saharan Africa, a region referred to as the “meningitis belt,” has a large proportion of meningitis cases. Epidemic meningococcal group A disease outbreaks have recorded incidence rates up to 100 per 100,000 (4). The introduction of MenAfriVac (Serum Institute of India Ltd, Hadapsar, Pune, India), a conjugate vaccine against serogroup A *N. meningitidis*, in sub-Saharan Africa has virtually eliminated Group A meningococcal meningitis outbreaks. However, new epidemics in Burkina Faso, Chad, Mali, Niger, and Togo with other serogroups (W and C) are now occurring (4). A systematic review of bacterial meningitis in Africa found that the most common pathogens were *N. meningitidis* (*n* = 2,433; 56%), *S. pneumoniae* (*n* = 1,758; 40%), and *Haemophilus influenzae* (*n* = 180; 4%).

Clinical outcomes vary geographically, with mortality rates ranging from 6% in Germany to 54% in Malawi (5, 6). Similarly, neonatal meningitis mortality also differs between developing countries (40–58%) and developed countries (10%) (7). Low-income countries have a significant incidence of bacterial meningitis and higher rates of survivors with long-term disabling sequelae. In a meta-analysis of 18,183 survivors of acute bacterial meningitis, the risk for a major neurological sequela as, motor deficit, bilateral hearing loss, cognitive impairment, visual impairment, hydrocephalus and, seizures, was highest in Africa (25.1%) and Southeast Asia (21.6%) than in Europe (9.4%) (8). This discrepancy between outcomes is most because survival and neurological sequelae depend on a rapid diagnosis and early treatment, both of which are difficult to have in resource-limited settings where laboratory support and antibiotic therapy are scarce.

Bacterial meningitis is still a prevalent, often undiagnosed, fatal infection in many African neonates, with a high death and morbidity rate. In many African healthcare settings, lumbar punctures are performed infrequently, and bacterial meningitis goes undiagnosed (9). Primary and secondary prophylaxis are equally necessary for reducing newborn infections. Improved prenatal, intrapartum, and postpartum care, exclusive breastfeeding, and the avoidance of low birth-weight infants are all likely helpful. Socioeconomic and maternal education significantly impact mother and newborn health and must be addressed to prevent neonatal meningitis (7). Also, several African nations with the most significant risk of neonatal death have been affected by conflict, war, or other natural calamities (10).

This chapter describes in detail the different pathogens commonly causing bacterial meningitis in Africa, their prevalence, pathophysiology, factors associated with pathogen entry into the brain, the interplay between the pathogen and the immune system of the central nervous system (CNS), and the consequences of this interplay. We also outline strides and recommendations in the diagnosis, management and prevention of each pathogen-caused meningitis.

## Neuroinflammation and role of microglia in bacterial meningitis

The pathophysiology of bacterial meningitis typically involves bacteria propagating into the brain through the bloodstream and then crossing the blood-brain barrier (BBB) however in a minor portion of cases, the bacteria enter directly through the cerebral tissue following skull fractures. Bacterial replication then occurs concurrently with the release of specific virulence factors, which trigger a cascade of signaling pathways that activate several transcription factors and initiate neuro-inflammatory processes that allow peripheral immune cells to enter the brain, causing BBB disruption. Thus, neuroinflammation, a process that should be a defensive mechanism, instead becomes dangerous for the host.

Bacterial infections of the brain are life-threatening because the brain is not easy to be reached by antibiotics because the BBB acts as a barrier between the brain and the systemic circulation (11). Nevertheless, death from bacterial meningitis does not occur because of the infection *per se*; the severe neuroinflammatory process that the host triggers in response to the infection results in the host's killer (12–15). Microglia are the resident immune sentinels of the brain with the primary function of eliminating invading pathogens by phagocytosis (12). Another function of microglial cells is to initiate a signaling process by releasing pro-inflammatory cytokines to recruit other immune cells, such as neutrophils, that reach the brain to help microglial cells in the process of pathogen elimination (12). Overall, neuroinflammation has a so-called “double-sword effect” even though the main scope of microglial pro-inflammatory response is for the host protection, the trafficking across the BBB of blood-borne immune cells usually causes severe disruption of the BBB with consequent intracerebral hemorrhage (12–19). Furthermore, activated microglia may secrete IL-1α, TNF-α, and C1q, generating reactive astrocytes known as A1; A1 astrocytes lose their capacity to support neuronal survival, outgrowth, synaptogenesis, and phagocytosis, and produces a neurotoxin that affects oligodendrocytes and can cause the neuronal death (20). In another study, newborn rats stimulated with LPS showed an increase in microglial cell activation in the hippocampus, cerebral cortex, and thalamus during their adulthood (21).

Microglia are very sensitive to external stimuli and can sense bacteria soon after they have invaded the brain (22, 23). Upon bacterial entry into the brain, microglia undergo a dramatic change in their morphology, and they are then classified as “activated” (23). Microglial activation occurs upon recognition by the microglial Toll-Like Receptors (TLRs) of specific pneumococcal components, such as peptidoglycan (PepG) and lipoproteins (LPPs) (24). PepG can cross the BBB, and it was recently reported that PepG originating from gut microbiota could modulate brain development (25).

When bacteria cross the BBB and invade the brain, microglia initiate their defensive action by eliminating the pathogens *via* phagocytosis (26). Endothelial cells line the internal walls of the blood vessels, including the BBB, and can release pro-inflammatory cytokines in response to bacterial components that can infiltrate the brain (27, 28). During bacterial growth, peptidoglycan (PepG) is cleaved and detached from bacterial cells and several proteins, such as lipoproteins (LPPs). A question that the scientific community still has not addressed is whether microglia can be activated when bacteria are not yet in the brain.

In the case of *E. coli*, its replication occurs concurrently with the discharge of bacterial products, including lipopolysaccharide (LPS), DNA, and other cell wall fragments inside the subarachnoid space (29), identified as pathogen-associated molecular patterns (PAMPs) (30, 31). These PAMPs are detected by pattern-recognition receptors (PRRs) and non-PRRs, both of which are essential immune system components (31, 32). The PRRs are classified into several families, including toll-like receptors (TLRs), nucleotide-binding oligomerization domain-like receptors (NOD)-like receptors (NLRs), C-type lectin receptors (CLRs), retinoic acid-inducible gene I (RIG-I)-like receptors (RLRs), and intracellular DNA-sensing molecules (30, 33), and the receptor for advanced glycation end products (RAGE), triggering receptors expressed on myeloid cells (TREM), and G-protein-coupled receptors (GPCRs) are examples of non-PRRs receptors (34). When immune receptors detect PAMPs, a cascade of signaling pathways is activated, promoting pro-inflammatory mediators. Cytokines, chemokines, and antimicrobial peptides are the mediators required to remove invading pathogens (35). During a meningitis infection, endogenous molecules released by stressed or damaged cells, known as damage-associated molecular patterns (DAMPs), activate the innate immune system by binding to PRRs and non-PRRs (30). The detection of PAMPs and DAMPs by immune receptors can result in adverse consequences, increasing the BBB permeability, allowing the peripheral immune cells to reach the cerebrospinal fluid (CSF), activating the glial cells triggering the neuroinflammation and long-term cognitive impairment in *E. coli* meningitis survivors (36, 37).

### Pneumococcal meningitis

#### Bacterial invasion of the brain through receptor-mediated transcytosis

The Gram (+) bacterium *S. pneumoniae* (pneumococcus) is the major etiological cause of bacterial meningitis worldwide (38, 39). The main route for pneumococci to reach the brain is the bloodstream; bacteria travel in the blood and easily reach the blood-brain barrier (BBB), a specialized vasculature system that separates the brain from the rest of the systemic circulation (38, 39). The brain is defined as “immune privileged” because of the presence of the BBB. The primary function of the BBB is to protect the brain from harmful substances that can enter the brain and cause cerebral damage (40). Pneumococci exploit the so-called receptor-mediated transcytosis to interact with the BBB and enter the brain tissue (38), a mechanism in which surface-exposed proteins can bind to specific receptors that are exposed on the plasma membrane of the endothelial cells of the BBB; this binding is the first and fundamental step of the process of bacterial passage across the BBB (38). Below are the mechanisms or virulence factors that the bacterium uses to cross the BBB.

#### The pilus-1 and RrgA

The pilus-1 is a “hair-like” structure on the surface of the bacteria and is presented in approximately 20-30% of pneumococcal strains (22, 41, 42). The pilus-1, particularly the tip protein RrgA present on top of the filament structure, was previously reported to significantly enhance the capacity of *S. pneumoniae* to bind to the BBB endothelium (41). Pneumococci use RrgA to bind to the platelet endothelial adhesion molecule 1 (PECAM-1), and the polymeric immunoglobulin receptor (pIgR) expressed on the brain's surface endothelial cells lining the internal wall of the BBB vasculature. Through this binding, pneumococci can enter the brain (43). Before being a pathogenic bacterium, *S. pneumoniae* is a commensal colonizer of the human nasopharynx, and most of the time, this colonization is completely asymptomatic (22).

#### Choline-binding protein A, also known as Pneumococcal surface protein C

CbpA, also known as PspC, is a surface-exposed protein anchored to the choline of the pneumococcal cell wall (44). CbpA was previously described to bind to the laminin receptor (LR), an essential molecule in cell adhesion to the basement membrane of brain endothelial cells (45). More recently, CbpA was also described to mediate the adhesion of pneumococci to the pIgR expressed by the BBB endothelium. Interestingly, the exact interaction between pneumococcal CbpA and pIgR also mediates the adhesion to the respiratory epithelium and colonization of the pneumococcus in the nasopharynx (43).

#### Neuraminidase A

NanA, a sialidase that cleaves sialic acids on host cells, helps pneumococci to penetrate the BBB and invade the brain (46). NanA was described to promote pneumococcal adhesion to and invasion of brain endothelial cells; furthermore, this interaction between pneumococcal NanA and the brain endothelium is enhanced by the sialidase activity of NanA (46).

#### Pneumococcal phosphoryl-choline

The first study that investigated receptor-mediated adhesion by Cundell and collaborators showed that bacterial phosphoryl-choline (ChoP) played a vital role in interacting with *S. pneumoniae* and human endothelial cells (47). PAFr was proposed to facilitate the interaction of pneumococci with the BBB endothelium, and some studies hypothesize a direct binding between pneumococcal ChoP with PAFr (48). On the other hand, others seem to point toward a more indirect role of PAFr in pneumococcal meningitis pathogenesis in which, during the inflammatory events that the host triggers the bacterial infection, PAFr is activated by the release of pneumococcal components that lead to the infiltration of immune cells, like neutrophils, into the brain (48). Such immune cell infiltration leads to openings within the BBB endothelium that facilitate the passage of pneumococci into the brain (48).

#### Pneumococcal interaction with neurons

Neurons are the main cellular component of the CNS and are responsible for transmitting electrical and chemical signals critical for all brain functions. Even though mortality due to bacterial meningitis is not dramatically high ranging from 10–30% globally (49–51), approximately 50% of survivors suffer from permanent neurological impairments, such as cognitive and motor disabilities and hearing loss, due to neuronal injury caused by the infection (52–54). The highest rates of bacterial meningitis worldwide belong to African children, and in almost one third of cases, *S. pneumoniae* is the etiological cause (55). Neuropsychological sequelae are frequently observed in African children that survive bacterial meningitis (55). Pneumolysin (Ply) is the pore-forming cytotoxin released by *S. pneumoniae* and can damage the host cells (56, 57). Generoso et al., have recently shown that the accumulation of pneumococci and toxic pneumococcal products, such as Ply, in the CSF compartments of the brain leads to neuronal damage with consequent dramatic impairment of neurological functions (58). Like it was previously described for other bacterial pathogens, pneumococci can exploit the interaction with the host cell cytoskeleton to invade neurons; neuronal cell death occurs due to cytoskeleton disruption (59, 60).

### Diagnosis, clinical presentation, and treatment

#### Management of pneumococcal meningitis today

Cure and prevention of infectious diseases are usually resolved with antibiotics and vaccines. Like other streptococcal infections, pneumococcal meningitis is routinely treated with β-lactam antibiotics (61, 62). Two main problems related to antibiotic treatment in managing bacterial meningitis are (1) β-lactam antibiotics have poor penetration of the BBB (63), (2) due to the indiscriminate use of antibiotics in the last decades, the problem of antibiotic-resistance is a constant threat to face in clinics (64); bacteria are highly-versatile microorganisms and can change in response to antibiotics, and new antibiotics can be discovered, but bacteria can rapidly adapt and develop resistance (65). Preventing is better than curing, and to build immunity toward pneumococcal infections, the introduction of pneumococcal conjugated vaccines (PCV) in the early 2000s has decreased the incidence of invasive pneumococcal disease, clinically defined as any type of infection caused by *S. pneumoniae* (66, 67). A decrease in admission rates was observed in Ethiopia among children affected with pneumococcal meningitis, yet several thousands of cases have been registered, which means that vaccination has still not yet provided significant protection to prevent the disease (68).

PCV is based on polysaccharides that compose the capsule surrounding the pneumococcal cell, and they are poorly immunogenic. There are more than 100 serotypes of *S. pneumoniae*, and all these serotypes are defined based on the polysaccharide composition. The current PCV (PCV13) is protecting only against 13 serotypes, therefore, we can build up a strong immunity only toward infections caused by the serotypes included in the PCV. The negative downstream effect of the introduction of PCV has been that the incidence of invasive pneumococcal disease caused by non-vaccine-types has increased (69, 70). The sub-Saharan region has by far the highest burden of acute bacterial meningitis in the world (71). In Malawi, 7 years after the introduction of PCV13 in 2014, a recent study has shown that non-vaccine serotype invasive pneumococcal disease, including meningitis, has increased (72). Furthermore, PCV has not significantly boosted the local immunity of the brain against pneumococcal infections; despite vaccination programs, hundreds of thousands of meningitis cases worldwide still occur yearly (49, 73).

#### Current treatments and clinical diagnosis

Pneumococcal meningitis is routinely treated in clinics with ceftriaxone, a “broad spectrum” cephalosporin (50). High concentrations of ceftriaxone in systemic circulation lead to increased penetration of the antibiotic through an inflamed BBB (63). To suppress excessive neuroinflammation, which leads to BBB endothelium breakdown and consequent brain edema and life-threatening hemorrhages, antibiotic treatment can be combined with the use of corticosteroids (74). Typical symptoms of suspected pneumococcal meningitis are high fever, stiff neck, nausea and vomiting, mental changes, intense headache, and sensitivity to light (75). Besides the classic tests performed after sampling which include bacterial culturing and microscopy detection of bacteria (50), more rapid diagnosis can be performed through immunochromatographic test (ICT), which can detect around 30% more pneumococcal meningitis cases than what usually caught with CSF culturing alone (76).

### New therapeutic and prophylactic approaches to cure and prevent pneumococcal meningitis

#### Blockade of host-pathogen interaction as an adjunct therapy to current antibiotics

It is important to have the availability of alternative therapies that can be used as adjunct treatments instead of antibiotics. Blood-borne pneumococci bind to PECAM-1 and pIgR expressed by brain vascular endothelial cells, and through this binding *S. pneumoniae* invades the brain (43). Iovino et al. have successfully shown that the administration of antibodies targeting PECAM-1 and pIgR *in vivo* significantly impairs pneumococcal invasion of the brain in mice, suggesting that the blockade of receptor-mediated adhesion and invasion can be a novel strategy to protect the brain from invading *S. pneumoniae* (43, 77). Bacterial adhesion to the BBB is only one step of a multi-event process during meningitis pathogenesis. Even though the blockade of bacterial interaction with the BBB can be achieved using meningitis animal models, the reality is much different because when patients are hospitalized with a diagnosis of bacterial meningitis, bacteria are unfortunately already in the CNS. In the brain, bacteria encounter neurons, and bacterial interaction with neurons causes severe and irreparable neuronal injury. Neuronal damage culminates into neuronal cell death, a pathological hallmark of all the impairments, so-called brain sequelae, which represent a dramatic issue in the burden of bacterial meningitis (52–54). Even if the bacterial infection is adequately cured, other eukaryotic cell neurons that have been damaged or killed by the bacteria cannot be replaced (78). For this reason, the World Health Organization (WHO) defines bacterial meningitis as a devastating disease. Recently, Tabusi et al. have shown a possible mechanism of neuronal cell death after pneumococcal infection. Using human neurons *in vitro* and a bacteremia-derived meningitis mouse model *in vivo*, they found that pneumococci use the cytoskeleton protein β-actin through the pilus-1 adhesin RrgA and the cytotoxin pneumolysin (Ply) to adhere to neuronal β-actin filaments and invade neurons. Interestingly, blocking this pneumococcal-β-actin interaction using antibodies reduced neuronal cell death (59). Can this be the beginning of a new neuronal protective therapeutic strategy?

#### Boosting the brain's immune response to protect the brain from bacterial infections

A well-orchestrated host-inflammatory response is crucial in eradicating infections from the brain; however, excessive or prolonged neuroinflammation can cause severe damage to the host (79). Microglia are the first responders to fight microbes and the main potentiators of neuroinflammation in the brain (12). Microglia reactive states are sometimes divided into pro-inflammatory M1-like “classically activated” and phagocytic M2-like “alternatively activated”; however, this classification is an oversimplification since microglial cells can present a large variety of functional phenotypes (80). The M1-like skewed microglial response is the typical hallmark of neuroinflammation in the pathophysiology of bacterial meningitis (11, 72). Activated microglia release various cytokines and chemokines and acquire migratory, proliferative, and phagocytic properties (12). Even before the infiltration of other immune cells from systemic circulation, the pro-inflammatory cytokines released by microglial cells pass through the BBB, increasing its permeability, and blood-borne leukocytes then have an easier access into the brain (17, 39, 81, 82). Leukocytes, like other immune cells that enter the brain, are huge cells in terms of size, and this continuous cellular trafficking soon leads to the rupture of the BBB vascular endothelium, which is life-threatening (9). Modulating microglial responses boosting the phagocytic capacity, and suppressing neuroinflammation could bring important advantages in managing bacterial meningitis (12).

## Meningococcal meningitis

*N. meningitidis* is an aerobic Gram (-) diplococcus species whose only host is human. It is found in the respiratory tract of healthy human beings but can cause devastating disease in those vulnerable. *N. meningitidis* is recognized as one of the three leading causes of meningitis in the world despite the presence of vaccines against almost five of its serotypes. Over 12 serotypes have been identified. However, only 5 of these have been identified to cause disease (10). *N. meningitidis* is grouped based on the surface polysaccharide capsule, and 13 meningococcal serotypes have been identified (A, B, C, D, 29E, H, I, K, L, Y, W-135, X, and Z). The majority of disease has been caused by A, B, C, Y, and W-135. Meningococcal disease in Europe and the Americas is mainly caused by serogroups B and C, whereas in Africa, the main causes are serogroups A and C. The capsule in serotype A is characterized by a non-sialic capsule with homopolymers of N-acetyl-D-mannosamine-1-P and (al-6) linked -N-acetyl-D-mannosamine-l-phosphate and has gene operon mynA-mynD. Sero-type C has a sialic acid capsule, homopolymers of sialic acid (a2-9)-linked- N-acetyl-neuraminic acid, and gene operon siaA and siaD (10).

### Pathogen characteristics and virulence factors

Virulence of *N. meningitidis* is hinged on several factors, including but not limited to capsule polysaccharide expression, expression of surface adhesive proteins (outer membrane proteins including pili, porins PorA and B, adhesion molecules Opa and Opc), iron sequestration mechanisms, and endotoxin (lipooligosaccharide, LOS) (10). In addition to these specific virulence factors, *N. meningitidis* has evolved genetic mechanisms resulting in high-frequency phase, antigenic variation, and molecular mimicry. Capsule switching, due to the allelic exchange of capsule biosynthesis genes by transformation, is one example that can allow the meningococcus to evade immune detection (83).

While *N. meningitidis* can be both capsulated or not, most strains isolated in blood or have almost always been capsulated. The capsule protects the bacteria against antibody/complement killing and inhibits phagocytosis (84).

### Pathogenesis and epidemiology

While *N. meningitidis* is an organism found in the nasal canal of healthy human beings, the rate of carriage and disease are variable and range from sporadic outbreaks, as seen in Europe, to epidemics in the African meningitis belt. To be able to survive, colonize and spread in a human being *via* the blood stream or CSF, the bacteria must harness specific properties, with its capsule being the main virulence factor, and its expression undergoes genetic regulation during pathogenesis. The capsule prevents cell adhesion and biofilm formation; thus, the expression needs to be downregulated or lost during carriage. However, the capsule is essential for survival in the blood and is thus upregulated during invasion into the bloodstream (85).

Adhesion to the mucosal membrane in the nasal canal is a key aspect of *N. meningitidis* pathogenesis. This is facilitated by the Type IV pili, which also play a key role in adhesion to endothelial cells, bacterial aggregation, twitching, motility, bacterial migration, and natural transformation (85, 86). The adhesion proteins then occur mediated by the opacity proteins, Opa, and Opc, with a typical integral membrane protein structure, which binds to carcinoembryonic anti-gen cell adhesion molecule (CEACAMs) receptor and extracellular matrix components (87). *N. meningitidis* has several adhesins enabling it to attach to several different receptors on the same target cell. It also means that it may respond differently or present differently during different stages of infection, thus mediating Neisserial adhesion to different cell types at different sites (86).

Following adhesion, the bacteria have to evade the complement system, which is the body's first line of defense. *N. meningitidis* achieves this through several mechanisms. The most studied is Factor H binding protein, which interacts with the Human Factor H, which is an inhibitor of the alternative complement pathway and therefore enhances the resistance of *N. meningitidis* while in serum to the complement system. There have been, however, other surface-exposed antigen components that have been found to inhibit the alternative complement pathway, thus suggesting that *N. meningitidis* has several mechanisms for evading the immune system, including NspA (*Neisseria* surface protein A), alkylated lipo oligosaccharide (LOS), and Neisserial heparin binding antigen (NHBA), which all play a role in the evasion of the complement pathway (88).

Capsular polysaccharides modulate several pathways of the complement cascade, further improving survival of *N. meningitidis* e.g., serogroup Y and W135 enhance activation of the AP by enhancing C3 activation and deposition, serotype B, C, W, and Y capsular polysaccharide have been found to inhibit complement pathway by inducing less C4b deposition, thereby limiting the antibodies' ability to mediate bacterial extermination (89).

Given these and more factors, the bacteria can enter the bloodstream and CSF while evading the complement system. Once it arrives in the blood, it then multiplies rapidly to infectious levels causing sepsis or translocating, crossing the BBB, and causing meningitis. The ability to cause invasive disease is dependent on environmental factors, meningococcal virulence factors, and lack of protective immune response. Certain factors like tobacco smoking, exposure to low humidity, and other co-infections increase the incidence of invasive disease (10).

The global incidence of *N. meningitidis* disease varies greatly by geographical distribution. Globally, the incidence is 500,000 to 1,200,000 worldwide, with over 50,000–135,000 deaths annually (90). The incidence in Europe ranges between 0.3–3.0 cases per 100,000, while in Africa, the incidence is 10–1,000 cases per 100,000 during pandemics in the meningitis belt. The categories most at risk for developing the invasive disease include newborns, children under the age of five, adolescents, immunocompromised, and the elderly (90).

### Diagnosis, clinical presentation, and treatment

Clinical presentation of *N. meningitidis* meningitis varies and may even appear benign but is characterized by sudden onset of high-grade fevers, headache, nausea, vomiting, unspecific rash, sore throat, and other upper respiratory tract infections. These symptoms can easily be confused with several other diseases, especially in areas of low incidence, and thus require that the clinician have a high index of suspicion as this type of meningitis will quickly progress to death. In the later course of the disease, it presents with neck stiffness, headache, photophobia, hemorrhagic or petechiae rash, altered mental state, and shock (91).

Early signs of sepsis, including tachycardia and hypotension, can be noted. A careful clinical examination should be performed with careful examination for a rash. The rash may initially appear as small papular, urticarial, or macular and later progress to petechia, purpura or ecchymoses, which are all early signs of thrombocytopenia, purpura fulminans, and DIC. The rashes can occur all over the body, but they are usually found on the palms and feet.

A positive Kerning's or Brudzinski sign (nuchal rigidity), fever, and altered mental status are the classical triad for a diagnosis of meningitis; however, these are rarely all present, and any two of fever, altered mental status, nuchal rigidity, and headache can be used to confirm the diagnosis of meningitis. Clinicians should consider *N. meningitidis* as the etiology if the patient presents with two of these plus a rash.

Purpura fulminans occurs when meningitis progresses further, due to vascular collapse initiated by LOS activating the release of inflammatory mediators characterized by cutaneous hemorrhage and skin necrosis due to vascular thrombosis. It can even lead to adrenal gland hemorrhaging and failure, termed Waterhouse-Freiderichsen syndrome and DIC; typically, the petechiae and erythema are seen on the skin but evolve into ecchymosis and later painful areas of necrosis with bullae and vesicles developing. Gangrenous necrosis may follow and lead to limb amputation and progression to DIC. Any evidence of bleeding from intravascular access, gingival bleeding, ecchymosis, or skin discoloration should be very concerning (92).

#### Diagnosis

The gold standard for diagnosis of *N. meningitidis* is by performing a lumbar puncture and collecting CSF for analysis. Based on patient history and clinical presentation, there needs to be a determination of whether there are higher chances of herniation prior to lumbar puncture. In a patient presenting with papilledema, seizures, and focal neurological symptoms, an LP may be delayed, and imaging, if available, done prior to the LP. CSF analysis should include Gram stain, protein, glucose, cell count, and protein count. Positive findings include increased opening pressure, pleocytosis of polymorphonuclear leukocytes, predominantly neutrophils, decreased glucose concentration, and increased protein levels. Gram stain may indicate Gram (-) diplococci; however, the gold standard for confirmation is CSF culture (93). Other tests could include PCR and latex agglutination to confirm *N. meningitidis*.

#### Treatment

Early recognition and initiation of treatment are vital in improving outcomes. Treating involves antibiotics, supportive care, coagulopathy management, contact tracing, and infection control.

Antibiotic treatment: Ceftriaxone and third-generation cephalosporins are generally preferable due to their high efficacy and easier dosing (94). Penicillin may also be used, and the dosing is 300,000 units/Kg/day IV or intramuscularly (IM), with a maximum dose of 24 million units per day. Penicillin is usually given as 4 million units every 4 h IV in adults and pediatric patients older than 1 month. High-dose penicillin is recommended for cultures with a sensitivity of penicillin minimum inhibitory concentration of 0.1 to 1.0 mcg/mL, although most clinicians will continue using third-generation cephalosporin instead (94). Dexamethasone dosing is 0.15 mg/Kg with a maximum dose of 10 mg every 6 h. This has no therapeutic benefit in meningococcal meningitis and, therefore, should be discontinued once this diagnosis is established. It is ideally administered 4 h prior to or concomitantly with antibiotics. It is not recommended if tuberculosis meningitis is suspected. Not recommended if meningococcemia with shock is suspected (94).

#### Vaccination

A lot of strides have been made in the prevention of meningococcal disease. Previously the main stay of the prevention of disease has been meningococcal polysaccharide vaccines; while these are inexpensive and more readily available, they are ineffective in infants and do not confer long-lasting immunity or provide herd immunity. Polysaccharide vaccines covering capsular groups A and C and A, C, W, and Y continue to play an essential role in emergency epidemic response in Africa. Also, recent reports indicate shortages of polysaccharide vaccines during NmC epidemic in Nigeria and Niger, presumably because a number of vaccine manufacturers are phasing out the production of polysaccharide vaccines (95).

Currently, several conjugate vaccines are available on the market including Menveo, Menactra, Meningitec, Menjugate, NeisVac-C, MenAfriVac, and MenHibrix, each targeting various strains. These have been used to address the different strains with MenAfric vaccine leading to almost near elimination of strain A where it has been implemented.

### *Escherichia coli* – Gram (-) meningitis

#### Pathogen characteristics and virulence factors

*E. coli* is a Gram (-) bacillus, facultatively anaerobic, and a commensal bacterium of vertebrates' gut, becoming an opportunistic pathogen in many intestinal and extra-intestinal infections. Pathogenic strains of *E. coli* are often categorized into pathovars, a group of bacterial strains that cause common illnesses and are designated using acronyms. The most common phenotypes identified are uropathogenic *E. coli* (UPEC), avian pathogenic *E. coli* (APEC), neonatal meningitis *E. coli* (NMEC) is the cause of newborns infection and newborn meningitis, extra-intestinal pathogenic *E. coli* (ExPEC), intestinal pathogenic *E. coli* (InPEC), Shiga toxin-producing *E. coli* (STEC), typical and atypical enteropathogenic *E. coli* (tEPEC and aEPEC); adherent-invasive *E. coli* (AIEC). There are also hybrid pathotypes in the InPEC pathovars, including enterohemorrhagic *E. coli* (EHEC) and enteroaggregative *E. coli* (EAEC) (96–100). *E. coli* pathovars are clonal groupings distinguished by serogroup, a distinct variety based on lipopolysaccharide (LPS, O antigen), and serotype based on a combination of O antigen (lipopolysaccharide), flagellar (H antigen), and capsular characteristics (K antigen) (101). The MNEC pathovar is the most common cause of Gram (-) neonatal meningitis, and the serotype K1 capsular antigen is approximately present in 80% of the *E. coli* isolate from neonatal meningitis (101, 102).

#### Pathogenesis and epidemiology

Cases of *E. coli* meningitis may be classified into different scenarios: neonatal meningitis, trauma and neurosurgery, and community-acquired/spontaneous meningitis. *E. coli* neonatal meningitis is a significant source of mortality and morbidity, with case fatality rates ranging from 5 to 25% and neurologic sequelae affecting 25 to 50% of survivors (103, 104). Symptoms and signs of *E. coli* bacterial meningitis in adults are headache, neck stiffness, and altered mental status. However, the typical meningitis trio of fever, neck stiffness, and altered mental state were found in 25% of subjects in an observational cohort study of individuals over 16. The mortality rate of *E. coli* meningitis in patients older than 16 ranged from 36 to 53% (105, 106), and 64% presented unfavorable outcomes (107).

**Figure 1:**
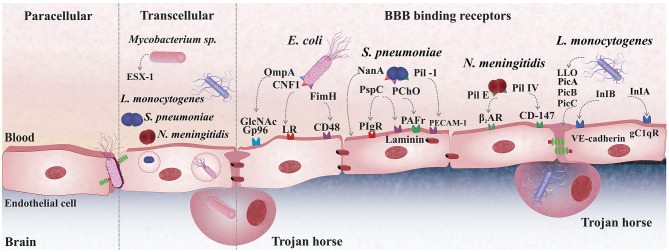
Bacterial mechanisms cross the blood-brain barrier (BBB). *S. pneumoniae* (pneumococcus) uses the pilus-1 and PspC (CbpA) to bind to PECAM-1 and pIgR expressed on the plasma membrane of brain endothelial cells; The PAF receptor plays also a role in pneumococcal adhesion to the BBB endothelium, although whether bacteria directly to it is still unclear; The laminin receptor (LR) mediates the passage of pneumococci from the basement membrane of the BBB endothelium promoting bacterial translocation into the brain. *E. coli* crosses the BBB *via* transcellular traversal or paracellular traversal. *E. coli* also binds with CD48 on brain microvascular endothelial cells *via* a type 1 fimbrial adhesion (FimH), outer-membrane protein A (OmpA) *via* N-acetylglucosamine (GlcNAc) or glucose-regulated protein-96 (Gp96), and cytotoxic-necrotizing factor 1 (CNF1) *via* the laminin receptor (LR). *L. monocytogenes* disrupts the phagosome membrane, releasing the phospholipases phosphatidylinositol-specific phospholipase C (Pic)-A and PIcB, as well as the toxin listeriolysin O. Internalin (Inl)-A and Inl-B bind to endothelial cells' receptors for the globular head of the complement component C1q (gC1qR) or VE-cadherin to cross the BBB. *Mycobacterium tuberculosis* infects brain tissue free or inside of macrophage, trojan-horse mechanism. The second mechanism is an active process that depends on an intact ESX-1, also known as type VII secretion systems, that induces phagosomal rupture in host phagocytes during transcellular migration. *N. meningitidis* uses Type IV pili and Opa, Opc binding proteins to adhere onto the mucocutaneous cells and uses Factor H to inhibit the alternative, complementary pathway.

*E. coli* colonizes the gastrointestinal mucosa and translocates from the lumen of the small intestine or colon into the systemic circulation before entering the CNS across the BBB (107). Oral administration of *E. coli* K1 resulted in steady and sustained gastrointestinal colonization in newborn rats in an experimental meningitis model (108). Using the same experimental model, *E. coli* K1 colonized the gut and crossed the gastrointestinal barrier. The newborn rodent acquired a fatal systemic infection with the bacterium in the blood circulation and brain tissue (109). *E. coli* K1 crosses the BBB by interacting with CD48 on brain microvascular endothelial cells *via* a type 1 fimbrial adhesion (FimH), outer-membrane protein A (OmpA) *via* N-acetylglucosamine (GlcNAc) or glucose-regulated protein-96 (Gp96), and cytotoxic-necrotizing factor 1 (CNF1) *via* the laminin receptor (LR) (110). Cytotoxic necrotizing factor (CNF1) is a bacterial virulence factor predominantly associated with meningitis-causing *E. coli* strains (111). This toxin helps *E. coli* K1 invade brain endothelial cells *in vitro*, and the bacteria crossed the BBB in a newborn experimental meningitis model. An isogenic mutant missing CNF1 was less invasive in brain endothelial cells and less able to enter the brain in the meningitis animal model (112). In human brain microvascular endothelial cell cultures, a double-knockout mutant with deleted OmpA and CNF1 genes was less invasive (113); for additional details, refer to Figure 1.

#### Consequences of the interplay between the pathogen and the immune system

*E. coli* LPS binds to TLR-4, and the adaptor molecule myeloid differentiation factor 88 (MyD88) interacts with the interleukin-1 receptor-associated kinase-4 (IRAK)-4. The IRAK then interacts with the receptor-associated factor (TRAF) family and connects to the TAK1 [Transforming growth factor-β (TGF-β)-activated kinase 1]/TAB1 (TAK1-binding proteins)/TAB2/TAB3 complex. TAK1 phosphorylates the NEMO (NF-κB essential modulator)/IKK (inhibitor of nuclear factor-κB (IκB) kinase)/IKK complex, which phosphorylates IKB, allowing the transcription factor NF-κB to be released and translocated to the nucleus (30, 114), which in turn activates several genes involved in the production of pro-inflammatory cytokines such as interleukin (IL)-1 beta, IL-6, tumor necrosis factor-alpha (TNF-α), and other inflammatory mediators (30, 105, 114). The flagella of *E. coli* and its protein flagellin binds to TLR-5, triggering the NF-κB and increasing the expression of the IL-8 chemokine. A preclinical study demonstrated that TLR-4 stimulation enhanced the phagocytosis of *E. coli* by microglial cells (115). Also, TLR-4 gene mutation was associated with an *E. coli* brain abscess in a twin pair of a newborn, according to a case report (116). Also, the MyD88-deficient animals could not prevent *E. coli* K1 neonatal meningitis, showing that MyD88 plays an essential role in early host defense (117). In mice, vulnerability to neurological morbidity changes intensely during the first few weeks of life. The neonatal brain is susceptible to infection leading to long-term neurological sequelae (118).

#### Diagnosis, clinical presentation, and treatment

##### Value of the neuroimmune changes in meningitis diagnostics and therapeutics

Significant rates of neurological morbidity and death continues to be associated with acute community-acquired bacterial meningitis. Differentiating between bacterial and viral meningitis remains a clinical challenge, particularly in individuals previously treated with antibiotics. Clinical studies and inflammatory biomarkers can help physicians with their diagnostic approach. The main characteristics of CSF bacterial meningitis, including *E. coli* meningitis, are the presence of polymorphonuclear (PMN) cells (>1,000 cells/L, 80–90% PMN), hyperglycorrhachia (40 mg/dL of CSF glucose, glucose CSF/blood ratio ~0.4 in children, and ~0.6 in neonates), and high CSF protein levels (>150 mg/dL) (119). In addition, CSF Gram stain allows rapid and accurate identification of bacteria in around 60–90% of samples, with a specificity of 97% or greater (120). However, the percentage of a positive Gram stain is partly dependent on the specific bacterial infection causing meningitis. Gram (-) bacilli had a Gram stain positive in approximately 50% of cases (121).

Other biomarkers, including lactate, C-reactive protein (CRP), and procalcitonin (PCT), are used to differentiate between bacterial and non-bacterial meningitis. A total of 236 infants with meningitis were included in a retrospective analysis. The infants with bacterial meningitis had 22.88% of positive CSF culture results. The levels of lactate dehydrogenase (LDH) and high sensitivity CRP (hsCRP) increased in the CSF of bacterial meningitis patients compared with non-bacterial meningitis patients. The positive microorganism culture was associated with higher levels of LDH and hsCRP in the CSF of the patients (122).

The determination of the pathogen in bacterial meningitis is not simple and is often associated with secondary infections. In a case report of meningitis caused by *E. coli*, the pathogen was detected only in blood and urine cultures with negative CSF culture (123). In some cases, it is not possible to identify the primary focus of *E. coli* due to the early use of antibiotics, therefore the polymerase chain reaction (PCR) in the CSF can be a useful test in patients who received antibiotic treatment before the lumbar puncture (121, 124). In general, CSF cultures may be negative even when bacterial meningitis is diagnosed (125, 126). Therefore, in addition to investigating secondary infections, it may be necessary to investigate other rare causes such as strongyloidiasis and chronic organ insufficiency before considering the *E. coli* infection as having been community-acquired (124). In addition, *E. coli* is identified in the CSF through studies involving these cultured bacteria in the bloodstream, which has helped clinicians infer the source of *E. coli* (127); once it becomes a major pathogen that causes bloodstream infection (128).

##### Therapeutics strategies

Although *E. coli* meningitis can be effectively treated with antibiotics, bacterial meningitis is especially severe in newborns and premature infants. However, the increased death rate happens in low- and middle-income nations where invasive infections are common; several patients do not have access to antibiotics (129), and the incidence of antibiotic resistance makes effective therapy a challenge (130). In neonates, 21 days of antibiotic is recommended for Gram (-) bacilli meningitis. Gentamicin should be added for infants and toddlers with diagnostic *E. coli* meningitis until CSF is sterile. Ampicillin-susceptible: ampicillin 300.0 to 400.0 mg divided in 4 to 6 doses can substitute cephalosporin. Ampicillin-resistant: Ceftriaxone 100.0 mg divided into two doses OR cefotaxime 200.0 to 300.0 mg divided into four doses PLUS gentamicin 7.5 mg divided into three doses. Length therapy, 21 days (131).

In conclusion, *E. coli* meningitis is a leading cause of death and morbidity globally, particularly among newborns. A patient with a high bacteremia rate is more prone to meningitis. For example, bacteremia with more than 103 colony-forming units (CFU)/mL of blood is often more going to result in meningitis than bacteremia with lesser CFU/mL of blood (101). The *E. coli* then translocates from the blood to the CNS, where it colonizes and causes meningitis. The inflammation causes further brain injury as well as long-term cognitive impairment. Pre-clinical models continue to further our understanding of the pathophysiology of *E. coli* meningitis and serve as a basis for developing new adjuvant and antibiotic treatments.

## Tuberculous meningitis

Tuberculosis (TB) continues to be a massive global health problem. Africa retains the second-highest TB burden at 25% of the global incidence in 2019 (120). Long-standing challenges to the eradication of the disease include the lack of effective vaccination, poverty, lack of education, poor access to early and effective health care, HIV/acquired immunodeficiency syndrome (AIDS), and the emergence of multidrug resistance (132). Extra-pulmonary TB constitutes 16% of total global TB notifications, tuberculosis meningitis (TBM) is the most serious form of extra-pulmonary tuberculosis and the most common form of neuro-tuberculosis, leading to death or severe disability in half of the affected individuals (133, 134).

### Pathogenesis and epidemiology

#### The systemic immune response to TB

TB is contracted through the inhalation of aerosolized mycobacteria tuberculosis (Mtb). The bacilli colonize pulmonary alveolar macrophages, which act as TB antigen-presenting cells to elicit an initial innate and consequent adaptive T-helper cell I (Th1) immune response (135). The inflammatory process encapsulates the infected cells in a granuloma and prevents the development of active disease in healthy individuals. However, in the very young, elderly, or immune compromised, the immune response may continue, resulting in active TB disease, destruction of the lung tissue, and potential dissemination of the TB bacillus to other organs (136, 137).

#### Dissemination to the CNS

TB dissemination commonly occurs hematogenously, and dissemination is often accompanied by miliary TB in children (138, 139). *In vitro* models have demonstrated that Mtb is able to invade the epithelial cells, replicate intracellularly, stimulate cell lysis and proliferate to neighboring cells (140). Further, Mtb is able to survive in infected macrophages and dendritic cells and may be transported out of the lungs to other organ systems (141), or it may invade and traverse vascular endothelial cells and be trafficked throughout the body in phagocytes (142). Host factors like polymorphisms in the genes encoding for antigen recognition and macrophage activation (143–145), perturbed pro-inflammatory cytokine release (146), and decreased vitamin D (133) may undermine the body's attempt to control the infection. Additionally, virulent TB strains may compromise the innate immune response, promote bacillary survival and replication, and cause more severe diseases like TBM (144, 147).

In the brain, Mtb can migrate across the protective BBB and blood-cerebrospinal fluid barrier (BCB) and enter the immune-limited domain of the CNS. *In vitro* and animal models have identified the Mtb gene Rv0931c (pknD) as a potential virulence factor that promotes CNS infection by enabling the bacilli to interact with extracellular factors on the brain endothelium leading to endothelial adhesion and rearrangement of the actin cytoskeleton of brain microvascular endothelial cells (142, 148). Another potential route of entry is the Trojan horse mechanism by which Mtb are trafficked across the BBB by infected macrophages (141).

### Diagnosis, clinical presentation, and treatment

Diagnosing TBM remains challenging due to the non-specific nature of its presentation and the lack of clinical, laboratory, or radiological tools to enable a swift and definitive diagnosis. Early diagnosis and commencement of treatment are considered the most important determinants of outcome (149). Most institutions treat patients on the presumed diagnosis of TBM based on a combination of clinical, laboratory, and radiological criteria in conjunction with laboratory tests. However, there is considerable variability across these criteria and testing platforms (150). To aid in uniform case definitions for research, a consensus statement was developed, which allows comparison between studies (151).

In the early phases of the disease, patients may present with non-specific sub-acute symptoms that are challenging to differentiate from those of benign conditions like an upper respiratory tract infection, particularly in children (152, 153). Headache, vomiting, weight loss or failure to thrive, meningism, and a decreased level of consciousness are among the most commonly presented symptoms (152, 154–161). A recent TB contact may be reported in 20–66% of cases (152, 155, 160, 161) and a previous history of TB in 13–27% of cases (162, 163).

CSF microscopy and chemistry are essential in the presumptive diagnosis of TBM. Common findings include elevated white cell count with lymphocytic predominance, low glucose and high protein (150). However, atypical findings are reported (164). The culture of Mtb in CSF was considered the gold-standard diagnostic test; however, CSF culture positivity yields are notoriously poor and can take more than 40 days due to the paucibacillary nature of CSF. In a recent review Bahr et al. report that in 22 adult TBM patients with confirmed TBM using Xpert^®^ MTB/RIF (Xpert; Cepheid, Sunnyvale, CA, USA), more than 90% had a low to very low bacillary burden (165). Consequently, diagnostic tests lack sufficient sensitivity; smear microscopy is 10–15% sensitive, and sensitivity on culture ranges between 50–60% (33). Although Cepheid's latest installment Xpert^®^ MTB/RIF Ultra (Ultra; Cepheid), is the currently recommended first-line diagnostic test for extrapulmonary TB, it's sensitivity for TBM ranges between 47.2–76.5% against the consensus criteria (34, 35) and in the region of 90% against culture (166). The performance improves with greater CSF volumes (which often are not accessible), and varies based on HIV co-infection (166, 167). It has therefore been suggested that diagnostic tests for TBM need to move beyond detecting the bacillus (165). Recently studies have examined the role of host inflammatory markers in both blood and CSF as possible diagnostic tools (162, 163). While these biosignatures demonstrate promise in small pediatric studies, larger studies that include adults and HIV co-infected patients are required.

#### Radiology

Radiological findings suggestive of TBM include basal and leptomeningeal enhancement, hydrocephalus, tuberculomas, and infarcts, and are an integral part of the presumptive TBM diagnosis (151). The characteristic feature of TBM is the presence of an inflammatory exudate in the basal cisterns of the brain (134, 163, 168). It consists of chronic non-necrotizing and necrotizing granulomatous inflammation. The predominant location of exudate at the base of the brain has several important implications; firstly, all the major cerebral vessels originate from the base of the brain and are at risk of being encapsulated by the exudate. Secondly, the accumulation of exudate in the basal cisterns interferes with the circulation of CSF, causing hydrocephalus. Thirdly, it envelopes and compresses the local cranial nerves, including the optic and oculomotor nerves resulting in cranial nerve palsies (134).

#### Vasculitis

Exudate coats all the major vessels in the Circle of Willis, with a predilection for the middle cerebral arteries, as well as their small perforators (163). This results in inflammation of the vessel wall, vascular occlusion, and vasospasm, which put the brain at significant risk of ischemia and infarction, commonly seen in the basal ganglia (169, 170).

#### Hydrocephalus

The presence of exudate in the basal cisterns blocks the flow of CSF around the upper brain stem and may occlude the cerebral aqueduct. This precipitates hydrocephalus and raised intracranial pressure, which adds to the risk of ischemia. TBM-associated hydrocephalus may be communicating if the obstruction to CSF flow occurs in the subarachnoid space or non-communicating when the flow is obstructed at the cerebral aqueduct or the outlet foramina of the fourth ventricle (171). Although non-communicating hydrocephalus occurs in a minority of cases (172), it can be fatal to perform a lumbar puncture in these patients. Determining the communicating nature of the hydrocephalus is crucial to safe patient management (171).

#### Tuberculomas and abscesses

Tuberculomas and TB abscesses may accompany TBM or occur independently. Tuberculomas are granulomatous, with a necrotic center surrounded by lymphocytes and epithelioid cells, which may merge to form Langhans giant cells. They are bordered by astrocytes and associated with edema and vascular proliferation (134, 173). TB brain abscesses are less common (174, 175). They comprise necrotizing granulomatous inflammation in the form of an encapsulated collection of pus containing Mtb bacilli. Both these lesions may arise after the initiation of anti-tuberculous treatment, sometimes termed a “paradoxical reaction” or be associated with immune reconstitution inflammatory syndrome (IRIS) in anti-retroviral treatment (ART) naïve TBM patients started on ART and anti-TB antibiotics in close succession. Neurological TB-IRIS can cause dramatic deterioration in patients and is of particular concern in resource-limited settings (176).

#### Spinal TB

Spinal TB may develop from TBM or secondary to vertebral TB (134). Exudate may be present along the meninges, cord and nerve roots leading to spinal arachnoiditis, intradural (extramedullary) tuberculomas or intramedullary tuberculomas, and tuberculous radiculomyelitis (134, 173). Exudate in the caudal sac can lead to dry lumbar taps or a high CSF protein (134, 173).

#### Antimicrobials

*Drug-*susceptible TBM in children is treated according to the WHO regimen with isoniazide (H: 10 mg/Kg, max. 300 mg), rifampicin (R: 15 mg/Kg, max. 600 mg), pyrazinamide (Z: 35 mg/Kg) and ethambutol (E: 20 mg/Kg) once daily for 2 months. This intensive phase is followed by a continuation phase of H and R daily for 10 months (177). However, following compelling recent data, in 2021, the WHO published an alternative, shorter 6 months' intensive regimen of HRZE for children and adolescents with drug-susceptible TBM (178). Drug susceptible TBM in adults is treated with: H (5 mg/kg, max. 300 mg), R (10 mg/Kg, max. 600 mg), Z (25 mg/Kg), and E (15 mg/Kg) once daily for 2 months. This intensive phase is followed by a continuation phase of H and R daily for 10 months (135). The penetration of R and E into the CSF is low (10–20% and 20–30%, respectively) but high for H and Z (80–90% and 90–100%, respectively) (135). The most common side effects of first-line tuberculostatic drugs are hepatotoxicity (H, R, Z), orange urine (R), peripheral neuropathy (H), and arthralgia (Z) (135). Isoniazid-resistant TBM is treated with REZ-levofloxacin (Lfx).

Multidrug-resistant (MD) TBM is treated with second-line tuberculostatics from group A (Lfx or moxifloxacin, bedaquiline, linezolid), group B (clofazimine, cycloserine), and group C (E, Z, delamanid, imipenem-cilastin or meropenem, amikacin, ethioniamide or prothionamide, para-aminosalicyclic acid) if needed, depending on the drug susceptibility testing of the infecting strain. Initially, a minimum of four drugs for 6 months is followed by three drugs, for a total of at least 18 months (135, 179). The penetration of either Lfx or moxifloxacin into the CSF is 70–80%, for ethioniamide, prothionamide, or cycloserine 80–90%, for linezolid 30–70%, and for amikacin 10–20% 5. P-aminosalicylic acid and E do not penetrate the CNS well and should not be counted on as effective agents for MDR-TBM. Amikacin penetrates the CNS only in the presence of meningeal inflammation. There are little data on the CNS penetration of clofazimine, bedaquiline or delamanid (180). The most common side effects of the fluoroquinolones (levofloxacin, moxifloxacin) include nausea, tremors, headache, and confusion. Myelosuppresion and optic neuropathy can be caused by linezolid. Cycloserine causes CNS depression leading to depression, seizures, and neuropathy. Concomitant administration of pyridoxine (vitamin B6) is advised when the patient is treated either with linezolid or cycloserine. Nephrotoxicity, and ototoxicity are the most common side effects of the aminoglycoside amikacin (135, 181).

#### Host-directed therapies: Corticosteroids

In HIV-negative adults and children with TBM, a Cochrane meta-analysis (updated in 2016; 9 trials; 1,337 patients; 469 deaths) showed that steroids reduce mortality by 25% (95% confidence interval: 13–35%) at 3–18 months of follow up. In one of the nine trials of 545 patients with follow up at 12 months, the effect on mortality was no longer apparent. There was no significant effect on disabling neurological deficits (182). The number of HIV-positive patients was too small to draw conclusions, but trials are currently being conducted on that group of patients (183). The WHO guideline advocates initial adjuvant corticosteroid therapy with dexamethasone or prednisolone tapered over 6–8 weeks (184). Studies investigating the expression of leukotriene A4 hydrolase (LTA4H), suggest that mainly HIV-uninfected patients with a high pro-inflammatory response (TT genotype) benefit from immune suppression by steroids (185). A tailored approach to immunosuppression based on genotype might improve overall outcomes in the near future.

#### Aspirin

Acetylsalicylic acid or aspirin is a non-steroidal anti-inflammatory drug, originally derived from willow tree leaves (Salix). Aspirin inactivates cyclooxygenase, inhibiting the synthesis of prostaglandin and thromboxane A2 in platelets. A low dose of aspirin impedes platelet aggregation, while its anti-ischemic and anti-inflammatory properties are dose-related.

Arterial ischemic stroke, prior to or during treatment, plays a major role in irreversible brain damage and poor outcome. Schoeman et al. compared low- (anti-thrombotic) and high-dose aspirin (anti-ischemic) with a placebo in young children with severe tuberculous meningitis (186). The study did not find differences in motor and cognitive function after treatment. The authors debated whether thrombosis plays a major role in cerebral infarction and hypothesized that proliferative vasculopathy (vasculitis) and vasospasm are the driving forces in TBM related cerebrovascular disease. A study in 98 HIV uninfected adults by Mai et al. also compared high and low-dose aspirin (both 2 months from the start of tuberculostatics and dexamethasone) with placebo. In the per-protocol analysis the aspirin group showed less stroke or mortality compared to the placebo (high dose: 11%, low dose: 15%, and placebo: 34%) (187). A trial in HIV-infected patients has been registered and is currently underway (188).

#### Thalidomide

Adjunctive corticosteroids reduce cytokine production and dampen subsequent inflammation, improving TBM survival but do not prevent morbidity. Therefore, additional immunomodulatory drugs are needed to improve TBM outcome. Thalidomide is known to reduce the TNF-α levels in CSF of children with TBM. The first safety study in 15 children with stage 2 TBM indicated that thalidomide was well tolerated in doses up to 24 mg/Kg/day (189). Unfortunately, the subsequent randomized trial in 47 children with stage 2 and 3 TBM needed to be terminated because of adverse events and deaths (13%) in the treatment arm of the study. Also, motor outcome and IQ after 6 months of anti-tuberculosis therapy was similar in the two groups. The above mentioned 13% mortality, however, was lower when compared with other described cohorts of such young (mean age of 4 years) and ill TBM patients, and the observed anti-inflammatory effects of thalidomide confirmed its possible treatment role for intracranial tuberculous mass lesions (190). With a lower thalidomide dose of 3–5 mg/Kg/day compared to the first safety study, children and adults with TBM complications such as cranial nerve palsies, blindness due to optochiasmatic arachnoiditis (2 months of treatment), and mass lesions (4 months of treatment) had a satisfactory clinical and radiological response without severe adverse effects (191). In summary, thalidomide may be considered for patients with developing large necrotizing TB abscesses, forms of spinal TB, TBM IRIS, and blindness caused by raised intracranial pressure or vasculitis compromising the optic chiasm (191).

#### Hydrocephalus management

Hydrocephalus management includes medical treatment, neurosurgical intervention, or a combination. Medical management includes repeated lumbar punctures and the use of acetazolamide and Lasix followed by neurosurgery if intracranial pressure does not normalize (171). Surgical options include external ventricular drains (sometimes used as a temporizing measure in severe cases), ventriculoperitoneal shunts and endoscopic third ventriculostomy (171). To date, there have been no clinical trials evaluating the ideal course of treatment and management practices remain heterogeneous (150).

#### Patient outcome

A meta-analysis on treatment outcomes of children with TBM demonstrated a mortality rate of 19.3%, and neurological sequelae in 54% of children who survived (192). Cognitive impairment, learning disabilities, emotional and behavioral problems, or motor impairment are common sequelae in these children (193). The poor neurodevelopmental outcome is associated with various factors such as young age, HIV infection, ethnicity, clinical severity, and delayed presentation and treatment (152). Early recognition and management of sequelae in children with TBM and support, availability, and access to appropriate care for them and their families, emphasizes the needs which are present in this population of patients (177). A meta-analysis on treatment outcomes of adults with TBM demonstrated a mortality rate of 24.7%, and neurological sequelae in 50.9% of adults who survived (194).

## Listeria monocytogenes

In the United States, *L. monocytogenes* accounts for 8% of all cases of bacterial meningitis, with the most common serotypes ½ b and 4 b (195). Preventive measures such as educational and awareness actions can be beneficial in the fight against bacterial meningitis. A study revealed that in the past 25 years there has been a decrease in the incidence of neonatal *Listeria sp*. meningitis possibly due to increased awareness of dietary restrictions for pregnant women (195).

Even though the incidence decreased, the rate of unfavorable outcomes among adults with *Listeria sp*. meningitis increased from 27 to 61%, with the emerging *L. monocytogenes* genotype sequence type 6 (ST6) identified as the main factor leading to poorer prognosis (196). A genomic sequencing study of these *Listeria sp*. strains identified a plasmid containing the benzalkonium chloride tolerance gene that was associated with decreased susceptibility to disinfectants commonly used in the food-processing industry. Strains containing the plasmid also had increased minimal inhibitory concentrations (MICs) to amoxicillin and gentamicin, two commonly used antibiotics in the treatment of *L. monocytogenes* (197).

Risk factors for developing *L. monocytogenes* include age over 60 years, chronic steroid recipients, alcoholism, immunosuppression, and malignancy (196–198). In a review of 820 cases of neurolisteriosis, the mortality rate was 26%; patients with seizures and age >65 were at even higher risk (198). In another study of 1,959 cases of listeriosis in France, risk factors for mortality included age >65 years, underlying disease, and focal listeriosis (199). In a recent prospective study of 818 cases of listeriosis in France, of which 252 were neurolisteriosis, factors associated with 3-month mortality were cancer, multi-organ failure, bacteremia, pre-existing organ dysfunction, monocytopenia, and adjunctive steroids (200). This is the first study to date showing an increase in mortality with the use of adjunctive dexamethasone in *Listeria sp*. meningitis (200). Adjuvant steroids should be stopped if *Listeria sp*. is found to be the cause of bacterial meningitis (201).

### Pathogenesis and epidemiology

*L. monocytogenes* is a food-borne pathogen that can cause gastroenteritis, bacteremia, meningitis, or maternal-neonatal infection (202). *Listeria sp*. has been isolated from water, sewage, dust, soil, and decaying vegetable matter (including silage and animal feed). Outbreaks of *Listeria sp*. infection have been associated with the consumption of contaminated coleslaw, raw vegetables, cheese, milk, contaminated turkey franks, cantaloupe, alfalfa tablets, diced celery, hog head cheese, and processed meats, thus pointing to the intestinal tract as the usual portal of entry (195). The largest outbreak was recently described in South Africa and accounted for 1,060 cases that were traced to processed meats (203). In addition, the infection can be transmitted from pregnant women to the neonate, since these women may harbor the organism in their genital tract and rectum and remain asymptomatic. Adults younger than 50 years who present with *Listeria sp*. meningitis should be screened for HIV infection (204).

Upon ingesting the bacteria, *L. monocytogenes* traverses the intestinal epithelium into the lamina propria and then disseminates to the liver, spleen, and brain (202). *L. monocytogenes* may cross the BBB by direct uptake from endothelial cells (202). Bacteria have been observed within endothelial cells, showing the ability of *L. monocytogenes* to invade cultured human brain microvascular endothelial cells, given that the listerial surface protein In1B is present. After entering cells, *Listeria sp*. utilizes listeriolysin O to escape from phagosomes by entering the cytoplasm (205). Once inside the cell, the resulting propulsion of *Listeria sp*. against the cell membrane facilitates that it can be phagocytosed by the adjacent cell, leading to further dissemination. *Listeria sp*. can also invade the CNS by transportation within leukocytes or *via* a neural route enabling cranial nerve invasion (202, 206).

### Diagnosis, clinical presentation, and treatment

*L. monocytogenes* meningoencephalitis can present with seizures and focal neurological deficits such as ataxia, cranial nerve palsies, or nystagmus secondary to rhombencephalitis (e.g., brainstem and cerebellar involvement) (194, 197–199). In an extensive review of neurolisteriosis (199), the most frequent clinical findings were fever, headache, and altered sensorium, with <50% having meningeal signs. In the MONALISA study, a total of 818 cases of listeriosis from France were identified, of which 252 (31%) were neurolisteriosis. The most common presentation was encephalitis (87%), with brainstem involvement in 17%. Clinical findings included nuchal rigidity (65%), aphasia (19%), seizures (18%), and focal limb weakness (12%) (200).

The mortality in neurolisteriosis remains high. In a study of 375 patients, the mortality rate was 31%, with age and concomitant bacteremia as independent prognostic factors (207). In the MONALISA study, the 3-month mortality rate was also 30%, and the most important predictors were ongoing organ neoplasia, multi-organ failure, aggravation of any pre-existing organ dysfunction, mechanical ventilation, monocytopenia, bacteremia, and administration of adjunctive dexamethasone (200).

#### CSF

In the MONALISA study, the median CSF WBC was 457 cells per μl, the median CSF protein was 2.1 g/L, and the CSF to blood glucose ratio was 0.31 (200). The CSF Gram stain and culture in *Listeria sp*. meningitis is only positive in 24–32% and 80–90% of patients, respectively (120, 194, 199). In the MONALISA study, the diagnosis was established by CSF culture in 84%, with the other 16% being documented by either CSF PCR (positive PCR in 63% of all patients) or by positive blood culture (200).

Often typical CSF findings predictive for bacterial meningitis might be absent and about 11–30% of patients with bacterial meningitis show negative CSF culture results. In patients with Listeria meningitis this percentage may be higher (51, 208–210). Other pathogen detection tools such as next-generation sequencing (NGS) have been successfully used to properly detect *L. monocytogenes* in a case whose clinical manifestations were suspected as tuberculous meningoencephalitis (211). Furthermore, the detection of a combination of specific biomarkers activated in the immune response in Listeria meningitis may help in the differential diagnosis (212). Finally, the use of a real-time PCR assay to detect and quantify *L. monocytogenes* DNA through specific amplification of the *L. monocytogenes* hly gene in CSF helped to improve the sensitivity of microbiological diagnosis in 214 samples from patients with suspected listeriosis (213). Furthermore, the sensitivity of the CSF Gram stain and culture decreases significantly in patients with prior antimicrobial therapy prompting the UK guidelines to recommend routinely obtaining a PCR for the two most common meningeal pathogens (*S. pneumoniae* and *N. meningitidis*) in patients presenting with meningitis (194, 214). Novel multiplex PCR assay panels are now widely used, incorporating several viral, bacterial, and fungal targets that also include *L. monocytogenes* with high sensitivity and specificity (215).

#### Antibiotic therapy

Third-generation cephalosporins are inactive for *L. monocytogenes* meningitis (194). In patients with suspected *L. meningitis* (e.g., neonates older than 50 days or with cellular immunodeficiency), ampicillin or penicillin G should be added (120, 194). An aminoglycoside should be added in patients with proven neurolisteriosis because of *in vitro* synergy, enhanced killing *in vivo*, and efficacy in animal models (120, 194).

In the MONALISA study, the addition of aminoglycosides was associated with reduced 3-month mortality in 679 patients with *Listeria sp*. bacteremia and or meningitis but not in the subset of patients with neurolisteriosis (200). Nevertheless, it is important to emphasize that a controlled clinical trial comparing ampicillin alone with ampicillin plus gentamicin has never been done in humans with listeriosis (194). An alternative agent in a penicillin-allergic patient is trimethoprim-sulfamethoxazole, which is bactericidal against *Listeria sp. in vitro* (192).

Trimethoprim-sulfamethoxazole was used in 17% of 679 patients with listeria bacteremia or meningitis and was associated with a reduction in 3-month mortality (199). Both vancomycin and chloramphenicol have been associated with an unacceptably high failure rate in patients with *Listeria sp*. meningitis and should be avoided (194). Carbapenems are active *in vitro* and in experimental animal models of *L. monocytogenes* meningitis and have been used in up to 3.4% of patients with listeriosis (194, 199). The fluoroquinolones and linezolid have good *in vitro* activity against *L. monocytogenes*, but there is a limited clinical experience (194).

#### Adjunctive corticosteroids

The routine use of early adjunctive dexamethasone has decreased mortality in adults in pneumococcal meningitis in high-income countries and is advocated by the Infectious Diseases Society of America, United Kingdom, and European guidelines (194). This benefit, unfortunately, has not been seen in studies in low-income countries from Africa or Asia, as patients present too late with advanced disease when the disease process is already established (120, 194). In a randomized, double-blind, placebo-controlled study from Malawi, there were no significant differences in mortality at 40 days (56% in the dexamethasone group vs. 53% in the placebo group) or when the analysis was restricted to patients with proven pneumococcal meningitis (53% in the dexamethasone group vs. 50% in the placebo group) (216). However, in this trial, approximately 90% of the patients were co-infected with HIV and had the advanced disease; the delayed presentation was also associated with a poorer outcome, although adjusting for this factor in the analysis had no effect. In patients with bacterial meningitis that is subsequently found not to be caused by *S. pneumoniae*, dexamethasone should be discontinued, especially if caused by *L. monocytogenes* or *Cryptococcus neoformans*, as steroids increase adverse clinical outcomes (120, 194, 199).

## Author contributions

All authors listed have made a substantial, direct, and intellectual contribution to the work and approved it for publication.
